# Combining Radar and Optical Sensor Data to Measure Player Value in Baseball

**DOI:** 10.3390/s21010064

**Published:** 2020-12-24

**Authors:** Glenn Healey

**Affiliations:** Department of Electrical Engineering and Computer Science, University of California, Irvine, CA 92617, USA; ghealey@uci.edu

**Keywords:** Bayesian, baseball analytics, machine learning, radar, intrinsic values, forecasting, sensors, batted ball, statistics, wOBA cube

## Abstract

Evaluating a player’s talent level based on batted balls is one of the most important and difficult tasks facing baseball analysts. An array of sensors has been installed in Major League Baseball stadiums that capture seven terabytes of data during each game. These data increase interest among spectators, but also can be used to quantify the performances of players on the field. The weighted on base average cube model has been used to generate reliable estimates of batter performance using measured batted-ball parameters, but research has shown that running speed is also a determinant of batted-ball performance. In this work, we used machine learning methods to combine a three-dimensional batted-ball vector measured by Doppler radar with running speed measurements generated by stereoscopic optical sensors. We show that this process leads to an improved model for the batted-ball performances of players.

## 1. Introduction

The expanded presence of sensor systems at sporting events has enhanced the enjoyment of fans and supported a number of new applications [1,2,3,4]. Measuring skill on batted balls is of fundamental importance in quantifying player value in baseball. Traditional measures for batted-ball skill have been based on outcomes, but these measures have a low repeatability due to the dependence of outcomes on variables such as the defense, the ballpark dimensions, and the atmospheric conditions [5,6]. The Major League Baseball (MLB) Statcast system [2] uses Doppler radar to measure parameters that include the initial speed and direction of batted balls. These parameters can be used to compute batted-ball statistics that are more repeatable than traditional statistics [7]. Research has shown that running speed is an important determinant of batter performance that is not measured by the radar sensor [8], but the Statcast system provides running speed data using stereoscopic optical sensors. This data provides the opportunity to improve the capability of batted-ball models by combining the radar measurements with the optical measurements. The objective of this study is to determine whether combining running speed measurements with batted ball measurements can be used to improve the accuracy of models for player performance.

Combining data from different sensors has been done successfully for numerous applications [9,10,11,12,13,14,15]. In this work, we employ a Bayesian framework and machine learning methods to build a model that combines radar batted ball data and optical running speed data. The approach generalizes a previous method [7] that considered lower-dimensional vectors consisting of only batted ball descriptors derived from a single sensor system. The model uses a nonparametric kernel method [16] to estimate the probability densities in Bayes law for vectors of radar and optical measurements acquired for over one hundred thousand batted-ball observations. A cross-validation process is used to find optimal smoothing parameters for the density estimates. The model utilizes the weighted on base average (wOBA) [17] linear weights model for run value. The result is the wOBA tesseract which represents a batted-ball value as a continuous function of four variables generated by the radar and optical sensors. Separate tesseracts are built to accommodate the effects of batter handedness. We present visualizations obtained by taking slices through the tesseracts to demonstrate properties of the model. We show that by including optical measurements for running speed, the new model is significantly more accurate than previous models that only consider measurements for batted-ball parameters.

## 2. Radar and Optical Sensors

**Figure 1:**
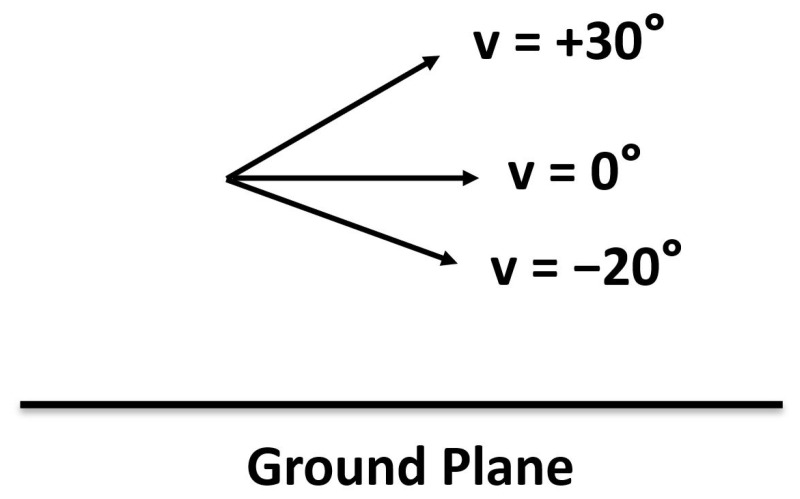
Vertical angle *v* where $v=0^{\circ }$ is parallel to the ground plane.

**Figure 2:**
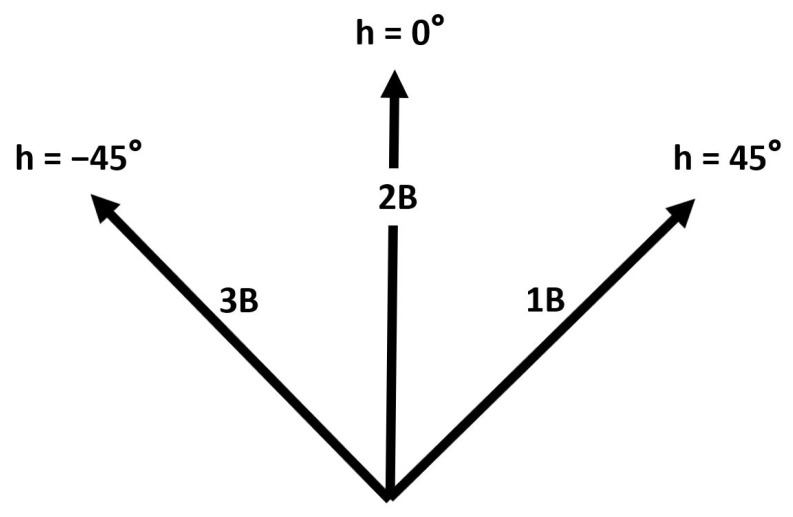
Horizontal angle *h* in the plane of the playing field where $h=-45^{\circ }$ is in the direction of third base (3B), $h=0^{\circ }$ is in the direction of second base (2B), $h=45^{\circ }$ is in the direction of first base (1B); the three rays intersect at home plate.

Beginning in 2017, the Statcast system employed radar along with optical stereo video sensors to acquire data for each MLB game. The trajectories of pitched and batted balls have been measured by Trackman’s phased-array Doppler radar component of Statcast. The Trackman radar is situated behind home plate and operates in the X-band at approximately 10.5 GHz. This radar system approximates the path of each pitch using a nine-parameter model defined by the pitch’s 3D acceleration which is assumed constant over the trajectory and the 3D velocity and position at a specified point. The system also measures the pitch spin rate from the distribution of Doppler shifts. In addition, the Trackman radar provides an estimate of the initial speed *s* and the 3D direction of batted balls. The direction is described by the vertical launch angle *v*, as shown in Figure 1, and the horizontal spray angle *h*, as shown in Figure 2. The angle *v* takes on values from $-90^{\circ }$ (straight down) to $+90^{\circ }$ (straight up) while the angle *h* takes on values from $-45^{\circ }$ (third base (3B) line) to $+45^{\circ }$ (first base (1B) line) for balls in fair territory.

The Trackman radar is well suited for tracking the ball, but the Doppler shifts from players are difficult to discern from returns from clutter due to the players’ slower speeds. For this reason, Statcast uses stereoscopic optical video from two arrays of cameras to track the movement of players. These arrays are usually positioned in the stands on the third base side of the field and are time synchronized with the radar. This allows the movement of defenders to be tracked which allows defensive skill to be quantified using measures such as reaction time, route efficiency, and speed. The combined optical and radar sensors can also be used to measure the time from batted ball contact until the batter reaches first base.

The success of a batter depends on both the quality of his batted ball contact as measured by the (s,v,h) vectors as well as his running speed as measured by time to first data. In this study we use Statcast radar and optical measurements from every regular-season MLB game during 2018. The data set includes (s,v,h) data for batted balls and associated time to first running speed measurements. For each batter with at least 20 ground balls, we use the average of his three fastest times to first to represent the batter’s time to first speed r. For switch-hitters who can bat both right and left-handed, a separate *r* value is computed using their batted balls as a right-handed batter and as a left-handed batter.

## 3. Learning the Model from Sensor Data

### 3.1. Bayesian Approach

Let *b* be a d-dimensional vector that can include the (s,v,h) batted-ball parameters and the *r* speed parameter. A batted ball can result in one of several outcomes $O_{j}$ such as an out or a home run. Bayes rule [18] can be used to compute the a posteriori probability of an outcome $O_{j}$ given *b* as

(1) $$P\left(O_{j}|b\right)=\frac{p\left(b|O_{j}\right)P\left(O_{j}\right)}{p(b)}$$

where p(b) and $p(b|O_{j})$ are the probability densities for *b* and *b* given $O_{j}$ respectively and $P(O_{j})$ is the a priori probability of outcome $O_{j}.$ We will derive a method that uses the a posteriori probabilities $P(O_{j}|b)$ to estimate the value of a batted ball given the vector *b* of sensor measurements.

### 3.2. Estimating the Conditional Densities

In order to compute the a posteriori probabilities $P(O_{j}|b)$ in Bayes rule we need to estimate the densities $p(b|O_{j})$ and p(b). The conditional densities $p(b|O_{j})$ have a complex dependence on the measurement vector b. An outcome $O_{j}$ of a single, for example, can occur for a slowly hit ground ball toward third base or a hard hit line drive to right field. Therefore we use a nonparametric technique known as kernel density estimation [19,20] to learn the densities. In this approach, we use a set of *n* sensor vectors $b_{i}$ to construct an estimate for p(b) according to

(2) $$\hat{p}\left(b\right)=\frac{1}{n}\sum_{i=1}^{n}G\left(b-b_{i}\right)$$

where G(·) is the Gaussian kernel

(3) $$G\left(b\right)=\frac{1}{{\left(2\pi \right)}^{d/2}{\left|\Sigma \right|}^{1/2}}\exp\left[-\frac{1}{2}b^{T}\Sigma^{-1}b\right]$$

where Σ is a diagonal covariance matrix defined by *d* parameters which determine the amount of smoothing for each element of the *b* vector.

### 3.3. Optimizing the Smoothing Parameters

The *d* diagonal elements of the matrix Σ play an important role in determining the accuracy of $\hat{p}\left(b\right)$ in Equation (2) [18]. If these smoothing parameters are too small then $\hat{p}\left(b\right)$ will be composed of spikes near the $b_{i}$ samples and if these parameters are too large then the resulting $\hat{p}\left(b\right)$ will be overly smooth. Cross-validation techniques have been developed to optimize the smoothing parameters by maximizing the likelihood of a set of $b_{i}$ vectors after building the estimate using other $b_{i}$ vectors [21]. An example of these techniques is leave-one-out cross-validation [16] in which the likelihood of each sample is computed after using the other samples to compute the kernel density estimate. We will take a similar but more efficient approach in this work to accommodate the size of our data set.

Let σ be the d-dimensional vector of diagonal elements of Σ. We partition the *n* measured $b_{i}$ vectors into an odd group and an even group depending on whether the vector was acquired in a game starting on an odd or even day of the month. Let $n_{v}$ be the smaller of the sizes of the two groups. The validation set $S_{O}$ is defined as the first $n_{v}$ vectors $b_{i}$ from the odd group and the validation set $S_{E}$ is defined as the first $n_{v}$ vectors $b_{i}$ from the even group. For set $S_{O},$ we find $\hat{p}\left(b\right)$ using the $n-n_{v}$ vectors $b_{i}$ that are not in $S_{O}$ as a function of the vector σ. The optimal σ for $S_{O}$ is defined as the vector $\sigma_{{}_{O}}^{*}$ that maximizes the pseudolikelihood [21,22] given by

(4) $$\sigma_{{}_{O}}^{*}=\underset{\sigma }{\arg\max}\prod_{b_{i}\in S_{O}}\hat{p}\left(b_{i}\right).$$

This process is repeated to find the vector $\sigma_{{}_{E}}^{*}$ that maximizes the pseudolikelihood for $S_{E}.$ The optimized smoothing vector $\sigma^{*}$ is found by averaging $\sigma_{{}_{O}}^{*}$ and $\sigma_{{}_{E}}^{*}.$

### 3.4. Computing Batted Ball Values

Each a posteriori probability $P(O_{j}|b)$ can be estimated using Bayes rule. The estimates for the densities p(b) and $p(b|O_{j})$ in Equation (1) are generated using Equations (2) and (3) where the model data for p(b) includes all *n* vectors $b_{i}$ and the model data for each $p(b|O_{j})$ is defined by the subset of the $b_{i}$ vectors with outcome $O_{j}.$ We use the optimized $\sigma^{*}$ smoothing vector derived using the method in Section 3.3 for each case. The a priori probabilities $P(O_{j})$ are estimated as $n_{j}/n$ where $n_{j}$ is the number of the *n* vectors $b_{i}$ with outcome $O_{j}.$ Using these estimates, $P(O_{j}|b)$ is computed using Equation (1).

Many statistics such as batting average, on-base percentage, slugging average, and on-base plus slugging have been defined to quantify offensive value [23]. Each of these statistics has certain deficiencies [17]. Batting average and on-base percentage, for example, assume that all hits such as singles and doubles are equally valuable. Slugging average overweights the value of extra-base hits (doubles, triples, home runs) compared to singles. On-base plus slugging places too much value on slugging average relative to on-base percentage. Weighted on base average (wOBA) [17] overcomes these deficiencies by weighting each possible outcome according to its run value. This property has made wOBA one of the most popular and useful offensive statistics [24].

Using wOBA each of the possible batted ball outcomes $O_{j}$ can be assigned a numerical value which allows the $P(O_{j}|b)$ probabilities to be used to compute a single expected value for b. This is implemented using wOBA by multiplying each outcome by its average run value $w_{j}.$ Thus, we can represent the expected value of a batted ball as

(5) $$\mathrm{wOBA}\left(b\right)=\sum_{j=0}^{5}w_{j}P\left(O_{j}|b\right)$$

where $O_{0}=$ out, $O_{1}=$ single, $O_{2}=$ double, $O_{3}=$ triple, $O_{4}=$ home run, and $O_{5}=$ batter reaches on error (ROE). The $w_{j}$ weights for MLB are compiled for each year at [25]. In this project, we process 2018 data for which the weights are $w_{0}=0.000$, $w_{1}=0.880$, $w_{2}=1.247$, $w_{3}=1.578,w_{4}=2.031$, and $w_{5}=0.920.$

If *b* is the three-dimensional vector b=(s,v,h) of batted-ball parameters, then the wOBA(b) function in Equation (5) can be represented by the wOBA cube. If *b* is the four-dimensional vector b=(s,v,h,r) of batted ball and running speed parameters, then the wOBA(b) function in Equation (5) can be represented by the four-dimensional wOBA tesseract. We will provide examples of the wOBA cube in this section and will analyze the wOBA tesseract in detail in Section 4.

**Figure 3:**
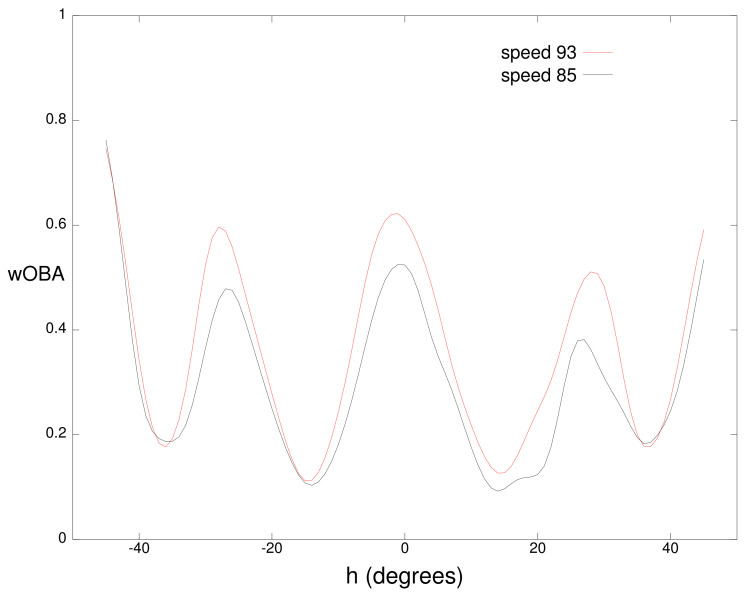
Weighted on base average (wOBA) for a batted ball with a vertical angle *v* of $-5^{\circ }$ for speed *s* of 85 miles per hour and 93 miles per hour.

**Figure 4:**
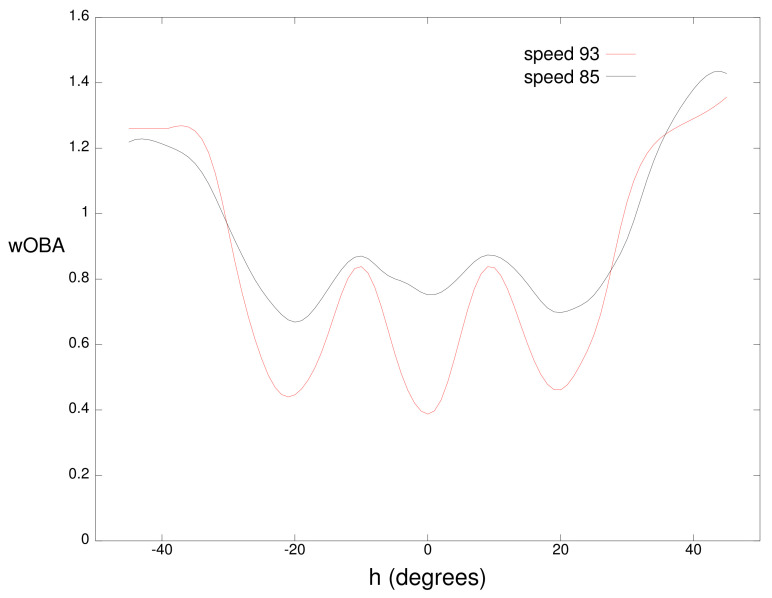
Weighted on base average (wOBA) for a batted ball with a vertical angle *v* of $16^{\circ }$ for speed *s* of 85 miles per hour and 93 miles per hour.

Figure 3 and Figure 4 examine one-dimensional slices through the wOBA cube. Figure 3 plots wOBA(b) for ground balls with a vertical angle of $-5^{\circ }$ that are hit at 85 and 93 miles per hour. Minima in the two curves correspond to the typical position of infielders with the minima from left to right corresponding to the third baseman, shortstop, second baseman, and first baseman respectively. Over most horizontal angles, balls hit at 93 mph have a higher value than balls hit at 85 mph since ground balls hit at a higher speed have a higher probability of eluding a defender.

Figure 4 plots wOBA(b) for balls hit in the air with a vertical angle of $v=+16^{\circ }$ at the same two speeds. Minima in these curves correspond to the typical position of outfielders with the minima near $-20^{\circ },0^{\circ },$ and $20^{\circ }$ corresponding to the left fielder, center fielder, and right fielder respectively. For this vertical angle, balls hit in the direction of an outfielder have a higher value for a speed of 85 mph because these balls often fall in front of the outfielder for hits while balls hit at 93 mph more frequently carry to the outfielder for outs. For both the ground balls and fly balls, the largest wOBA values occur for balls hit near the foul lines ($\left|h\right|=45^{\circ }$) which often result in extra-base hits instead of singles.

**Figure 5:**
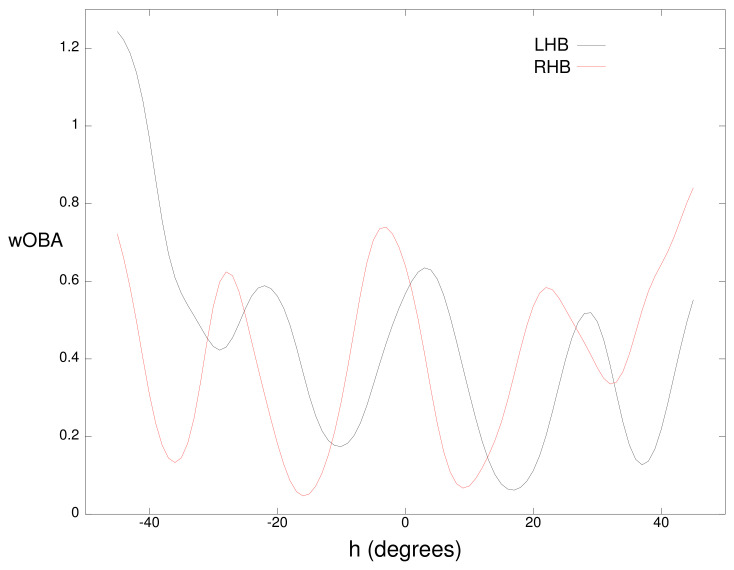
Weighted on base average (wOBA) for a batted ball with a vertical angle *v* of $-5^{\circ }$ and a speed *s* of 93 miles per hour for left-handed batters (LHB) and right-handed batters (RHB).

Fielder positioning is dependent on whether a batter is right-handed or left-handed. For this reason, we partition the measured *b* vectors by batter handedness and learn two separate wOBA(b) functions: wOBAl(b) for left-handed batters and wOBAr(b) for right-handed batters. As an example, Figure 5 plots wOBAl(b) and wOBAr(b) as a function of the horizontal angle *h* for a batted ball with a vertical angle *v* of $-5^{\circ }$ and a speed *s* of 93 miles per hour. Each curve has four minima which correspond to the typical location of the four infielders. Each of these typical locations is shifted a few degrees to the left for right-handed batters due to fielder positioning. The value of wOBAl(b) or wOBAr(b) will be referred to as the intrinsic value of the batted ball.

### 3.5. Player Statistics

A player’s performance on batted balls is measured by statistics that are compiled over a period of time. Each batted ball can be assigned the weight $w_{j}$ based on its outcome as described in Section 3.4. This outcome-based value depends on variables such as the defense, the atmospheric conditions, the ballpark dimensions, and random noise which are independent of batter skill. Let *O* denote the average of a player’s outcome-based values on batted balls over a period of time. The statistic *O* is also known as wOBA on contact or wOBAcon. A player’s intrinsic values are based on parameters (s,v,h,r) that a player has direct control over. The average of these intrinsic values over time has been shown to have a significantly higher degree of repeatability than the average *O* of the outcome-based values [7]. We refer to the average of a batter’s intrinsic values computed using the three-dimensional vector b=(s,v,h) of batted-ball parameters as $I_{3}$ and we refer to the average of a batter’s intrinsic values using the four-dimensional vector b=(s,v,h,r) that also includes his time to first estimate *r* as $I_{4}.$

## 4. wOBA Tesseract

In previous work [8] we showed that players who outperform their $I_{3}$ wOBAcon estimate tend to be faster runners, and many players who underperform their $I_{3}$ are slower runners. This motivates augmenting the wOBA cube with batter running speed to generate the wOBA tesseract.

### 4.1. Time to First Measurements

The Statcast system generates multiple measurements of running speed. Statcast measures sprint speed, which is derived from a runner’s fastest one second window on individual plays, and time to first which measures the time from batted ball contact to when the batter touches first base. For our application we use time to first, which includes factors such as a batter’s time to recover from the swing and start initial acceleration which affects his ability to beat out a hit.

**Figure 6:**
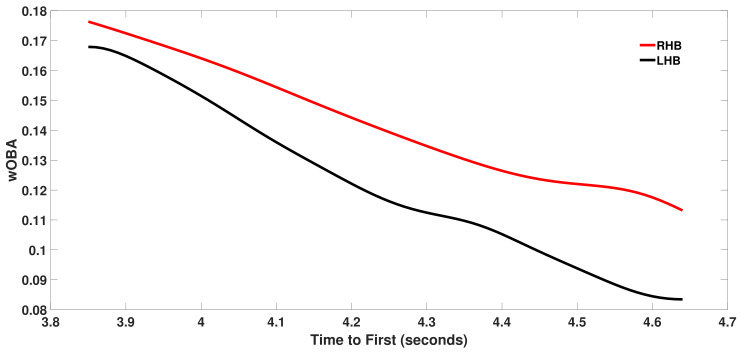
Weighted on base average (wOBA) versus time to first (r) in seconds over all batted balls with a vertical angle $v<10^{\circ }$ for right-handed batters (RHB) and left-handed batters (LHB) in 2018.

As described in Section 2, we define the running speed parameter *r* for batters with at least 20 ground balls as the average of the player’s three fastest measured times to first. For switch-hitters a separate *r* value is computed for plate appearances as a right-handed and as a left-handed batter. All other things being equal, we would expect left-handed batters to have smaller *r* values because they start closer to first base. For the 2018 season, the average *r* value over 207 qualifying left-handed batters was 4.245 s and the average *r* value over 319 qualifying right-handed batters was 4.305 s. Table 1 and Table 2 present the left-handed and right-handed batters with the fastest *r* values for 2018. Figure 6 plots wOBA as a function of *r* for right-handed and left-handed batters for all batted balls with a vertical angle of less than 10 degrees in 2018. These are ground balls for which the *r* value is most relevant. We see that there is a strong dependence of batted ball value on running speed as wOBA decreases as *r* increases. We also see that right-handed batters have a higher wOBA for a given *r* since a higher fraction of ground balls from RHB are hit to the left side of the infield which requires a longer throw to first base.

### 4.2. Tesseract Examples

**Figure 7:**
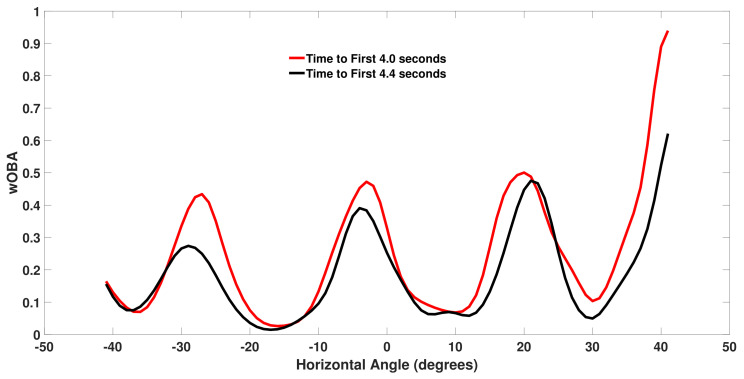
Weighted on base average (wOBA) for right-handed batter (RHB) batted balls with a speed *s* of 87 miles per hour and a vertical angle *v* of $-9^{\circ }$ for two time to first (r) values.

**Figure 8:**
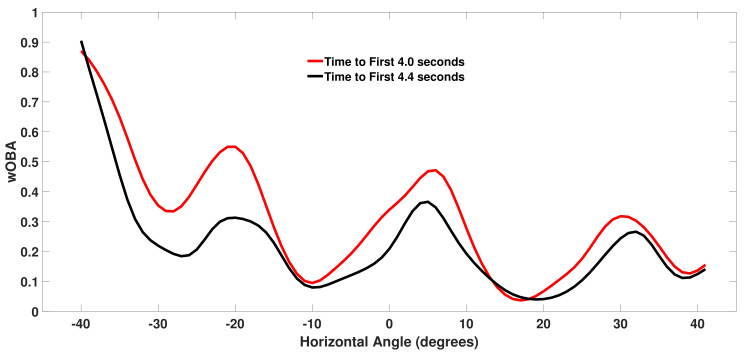
Weighted on base average (wOBA) for left-handed batter (LHB) batted balls with a speed *s* of 97 miles per hour and a vertical angle *v* of $-12^{\circ }$ for two time to first (r) values.

The wOBA tesseract defines the mapping from (s,v,h,r) to intrinsic value. A separate wOBA tesseract was generated for right-handed and left-handed batters by applying the process described in Section 3 to 63,301 batted ball and time to first measurements for right-handed batters and 44,247 measurements for left-handed batters acquired during the 2018 MLB regular season. Figure 7 and Figure 8 provide examples of slices through the tesseract.

Figure 7 plots wOBA(b) for right-handed batters for two different values of *r* as a function of the horizontal spray angle *h* with the initial batted ball speed and vertical launch angle fixed at s=87 mph and $v=-9^{\circ }.$ The red curve corresponds to a faster than average time of r=4.0 seconds and the black curve corresponds a slower than average time of r=4.4 seconds. The four minima in the curves correspond to the typical position of the four infielders against right-handed batters. Near these minima we have a ground ball hit directly at an infielder and the wOBA values are similar for the different values of r. As we move away from the minima we see that a faster runner (red curve) tends to produce a higher wOBA. We see that the largest wOBA values are observed for ground balls hit near the first base line as this horizontal angle is often undefended against right-handed batters and balls down the line may go for extra bases.

Figure 8 plots wOBA(b) for left-handed batters for two different values of *r* as a function of the horizontal spray angle *h* with the initial batted ball speed and vertical launch angle fixed at s=97 mph and $v=-12^{\circ }.$ The red curve corresponds to a faster than average time of r=4.0 seconds and the black curve corresponds a slower than average time of r=4.4 seconds. The four minima in the curves correspond to the typical position of the four infielders against left-handed batters. We see that the minima are shifted to the right compared to the minima for right-handed batters shown in Figure 7. Near three of these minima the wOBA values are similar for the different values of r. For a ground ball hit directly at the third baseman near $h=-28^{\circ }$, a faster runner enjoys an advantage since the third baseman will often be playing shallower to defend against a bunt for the faster runner and a 97 mph ground ball has a better chance of resulting in a hit. As we move away from the minima we see that a faster runner (red curve) tends to produce a higher wOBA. We see that the largest wOBA values are observed for ground balls hit near the third base line as this horizontal angle is often undefended against left-handed batters and balls down the line may go for extra bases.

### 4.3. Comparing $I_{3}$ and $I_{4}$

We computed the $I_{3}$ (wOBA cube) and $I_{4}$ (wOBA tesseract) estimates of wOBAcon for all batters in 2018 with at least 250 balls in play. Table 3 is a list of the $I_{3}$ leaders. These batters are known for their high quality of contact. Table 4 is a list of the $I_{4}$ leaders which factors running speed in addition to quality of contact into the value of each batted ball. We see that several of the slower runners (Gallo, Martinez, Judge, Goldschmidt) have a lower $I_{4}$ than $I_{3}$ while several of the faster runners (Trout, Story, Yelich, Betts) have a higher $I_{4}$ than $I_{3}.$ The value of $I_{4}-I_{3}$ depends on both the batter’s running speed parameter *r* and his particular collection of batted balls.

Table 5 is a list of the batters with the highest $I_{4}-I_{3}$ for 2018. These are the batters that would be expected to have the largest gain in wOBAcon due to their running speed given their collection of batted balls. We see that all of these players have better than average values of the running speed parameter r. Note that for switch hitters two values (L/R) of *r* are used.

Table 6 is a list of the batters with the lowest $I_{4}-I_{3}$ for 2018. These are the batters that would be expected to have the largest loss in wOBAcon due to their running speed parameter *r* given their collection of batted balls. We see that all of these players have worse than average values of r.

### 4.4. Variance Reduction

Differences between a batter’s observed wOBAcon *O* and his $I_{3}$ are due to several factors including running speed, susceptibility to shifts, the ballpark, the weather, and random noise. By developing the $I_{4}$ statistic we improve the accuracy of the estimate by explicitly modeling the dependence of each batted ball on the running speed parameter r.

Table 7 is a list of the batters with at least 250 batted balls with the highest $O-I_{3}.$ We see that each of these batters had a faster than average running speed r. In addition, several of these batters, such as Carlos Gonzalez and Trevor Story in Colorado, benefited from their home ballparks [6]. We see that in each case the use of the wOBA tesseract to generate $I_{4}$ improved the accuracy of the model as $O-I_{4}$ is less than $O-I_{3}$.

Table 8 is a list of the batters with at least 250 batted balls with the lowest $O-I_{3}.$ We see that each of these batters had a slower than average running speed *r* except Joe Panik who was slightly better than average. Several of these players (Morales, Moreland, Calhoun, Martinez, Carpenter) were shifted on during a large fraction of their plate appearances. We see that in each case the use of the wOBA tesseract to generate $I_{4}$ improved the accuracy of the model as $|O-I_{4}|$ is less than $|O-I_{3}|.$

If we consider all of the players with at least 250 batted balls in 2018, the R-squared for the set of points $\left(O,I_{3}\right)$ is 0.79 and the R-squared for the set of points $\left(O,I_{4}\right)$ is 0.85. Therefore, the model that includes running speed using the *r* parameter has increased accuracy for representing a batter’s wOBAcon. We therefore expect that $I_{4}$ is a better estimate of wOBAcon skill and provides more value for projection [7].

## 5. Discussion

Player valuation is a critical task for professional baseball teams that operate in an environment where player contracts are frequently worth tens of millions of dollars. Many statistics have been developed to quantify the offensive value of players. During the twentieth century these statistics, for example batting average, on base average, and slugging percentage, were based on outcomes such as whether the offensive player got a hit or made an out [23]. These outcomes, however, depend on many variables that are beyond the control of the offensive player such as the opponent fielders, the ballpark dimensions, and the weather. This dependence reduces the reliability of these statistics. The use of outcomes has also made it difficult to separate the impact of the key components that contribute to offensive value: batting skill and running speed. There have been some attempts to isolate the contributions of these components. For example, researchers have attempted to quantify running speed by using metrics like the Bill James speed score [26] which is based on factors that include an offensive player’s number of triples and stolen base attempts. But such a measure depends on factors besides running speed namely a player’s power-hitting ability and how often his team’s manager calls for stolen base attempts.

Starting with the PITCHf/x system [27], sensors have been available in all MLB ballparks to recover the 3D trajectory of pitched balls since 2008. The collection of sensors has evolved and expanded and the current system, Statcast [2], consists of multiple sensor types that collect seven terabytes of data during each MLB game. Large sets of sensor data provide benefits for measurement especially in the ability to reduce the variance of estimators [28]. In addition, sensor data has enabled the discovery and measurement of new skills. Pitch trajectory data, for example, uncovered the large role that a catcher plays in determining the probability that a pitch is called a strike. This led to the quantification of a new skill called pitch framing [29] that is highly valued in the sport. Sensor data has also led to advances in the quantification of defense [30] and pitch sequencing [31]. The measurement of batted ball vectors has enabled the calculation of batting statistics that are more reliable than statistics that depend on outcomes [7]. The ability to measure running speed enables new insights into how different skill components affect offensive performance. New sensor systems [32] are becoming available that measure biomechanical data for batters and pitchers which will increase understanding of how players achieve given levels of performance [33]. These measurements can also be used to improve the level of detail of models for predicting the result of matchups [34,35].

The ability to derive models from large sets of sensor data has been enhanced by recent advances in machine learning methods [36,37,38]. The discrete nature of baseball makes its analysis highly amenable to these methods [39]. For many applications [40,41] the use of nonparametric models enables the recovery of functions with a complex dependence on a set of variables. In this work, we use nonparametric density estimates [16] in a Bayesian framework [18] to model a player’s offensive performance using batted ball vectors and running speed measurements generated by radar and optical sensors. We show that by applying machine learning methods to a large set of measurements acquired by multiple sensors we obtain a model with significant advantages over previous models for representing a player’s offensive performance.

## 6. Conclusions

Analytical models in baseball have proven valuable for applications involving strategy [17,31,34,35], player development [33], and player evaluation [42,43]. We have combined data acquired by radar and optical sensors to generalize the 3D wOBA cube to the 4D wOBA tesseract. The new model accounts for the impact of batter running speed and is significantly more accurate than previous models. Thus, the use of multiple sensors enables the generation of a model that is more accurate than the model that is obtained by using either sensor in isolation. This accuracy enables the computation of offensive statistics that more reliably assess talent level on batted balls and support more accurate projections of future performance. This approach also allows separation of the impact of batted-ball skill and running speed in offensive value. An important advantage of this separation is that each skill can be regressed and projected using individual reliability and aging curves before conversion to projected offensive value during forecasting [44]. The wOBA tesseract also has the potential to improve defensive metrics by quantifying the relationship between the batter’s running speed and the difficulty of a play. We have shown that the wOBA tesseract enables visualizations that provide insights into the mapping between batted-ball and running speed parameters and intrinsic value. The process of combining sensor data and machine learning techniques to generate new statistics can be readily adapted to support other areas of sports analytics.

## Figures and Tables

**Table 1:** Fastest time to first (r) for left-handed batters (LHB) in seconds, 2018.

LHB	Time to First (r)
Dee Gordon	3.807
Billy Hamilton	3.814
Roman Quinn	3.824
Magneuris Sierra	3.836
Cody Bellinger	3.879
Jack Shuck	3.882
Brett Gardener	3.909
Mallex Smith	3.929

**Table 2:** Fastest time to first (r) for right-handed batters (RHB) in seconds, 2018.

RHB	Time to First (r)
Delino DeShields	3.855
Dansby Swanson	3.884
Trea Turner	3.896
Jose Altuve	3.896
Harrison Bader	3.899
Starling Marte	3.904
Scott Kingery	3.923
Adam Engel	3.929

**Table 3:** Weighted on base average (wOBA) cube $\left(I_{3}\right)$ leaders for 2018.

Batter	$I_{3}$
Joey Gallo	0.597
Aaron Judge	0.544
Julio Martinez	0.544
Mike Trout	0.541
Paul Goldschmidt	0.531
Matt Carpenter	0.527
Giancarlo Stanton	0.524
Christian Yelich	0.522

**Table 4:** Weighted on base average (wOBA) tesseract $\left(I_{4}\right)$ leaders for 2018, difference between wOBA cube and wOBA tesseract values $(I_{4}-I_{3}),$ and time to first (r) in seconds.

Batter	$I_{4}$	$I_{4}-I_{3}$	Time to First (r)
Joey Gallo	0.589	−0.008	4.319
Mike Trout	0.542	+0.001	4.062
Julio Martinez	0.535	−0.009	4.340
Aaron Judge	0.534	−0.010	4.487
Trevor Story	0.529	+0.015	3.955
Christian Yelich	0.527	+0.005	4.080
Mookie Betts	0.526	+0.007	4.055
Paul Goldschmidt	0.522	−0.009	4.309

**Table 5:** Largest differences between weighted on base average (wOBA) cube and wOBA tesseract values $\left(I_{4}-I_{3}\right)$ for 2018 and time to first (r) in seconds; two *r* values are given for switch-hitters.

Batter	$I_{4}-I_{3}$	Time to First (r)
Cody Bellinger	0.025	3.879
Ozzie Albies	0.022	3.936/3.942
Niko Goodrum	0.019	4.08/4.022
Rougned Odor	0.018	3.984
Dansby Swanson	0.018	3.884
Odubel Herrera	0.017	3.969
Scott Kingery	0.017	3.923
Brandon Nimmo	0.017	4.113

**Table 6:** Smallest differences between weighted on base average (wOBA) cube and wOBA tesseract values $\left(I_{4}-I_{3}\right)$ for 2018 and time to first (r) in seconds; two *r* values are given for switch-hitters.

Batter	$I_{4}-I_{3}$	Time to First (r)
Yasmani Grandal	−0.035	4.663/4.966
Victor Martinez	−0.034	4.634/4.965
Kendrys Morales	−0.031	4.788/4.816
Justin Bour	−0.029	4.498
Chris Davis	−0.027	4.491
Albert Pujols	−0.025	4.839
Yangervis Solarte	−0.022	4.556/4.649
Joey Votto	−0.022	4.575

**Table 7:** Largest differences between observed weighted on base average (wOBA) on contact (O) and wOBA cube values $\left(O-I_{3}\right)$ for 2018; differences between *O* and wOBA tesseract values $(O-I_{4});$ and time to first (r) in seconds; two *r* values are given for switch-hitters.

Batter	$O-I_{3}$	$O-I_{4}$	Time to First (r)
Carlos Gonzalez	0.063	0.054	4.150
Ronald Acuna	0.051	0.039	3.945
Mallex Smith	0.050	0.039	3.929
Brandon Nimmo	0.049	0.033	4.113
Chris Taylor	0.048	0.039	4.017
Trevor Story	0.045	0.030	3.955
Eddie Rosario	0.045	0.029	3.969
Yoan Moncada	0.045	0.029	4.094/4.175

**Table 8:** Smallest differences between observed weighted on base average (wOBA) on contact (O) and wOBA cube values $\left(O-I_{3}\right)$ for 2018; differences between *O* and wOBA tesseract values $(O-I_{4});$ and time to first (r) in seconds; two *r* values are given for switch-hitters.

Batter	$O-I_{3}$	$O-I_{4}$	Time to First (r)
Kendrys Morales	−0.064	−0.033	4.788/4.816
Mitch Moreland	−0.063	−0.052	4.262
Kole Calhoun	−0.058	−0.045	4.315
Nelson Cruz	−0.055	−0.049	4.395
Albert Pujols	−0.054	−0.029	4.839
Victor Martinez	−0.052	−0.018	4.634/4.965
Matt Carpenter	−0.048	−0.037	4.281
Joe Panik	−0.047	−0.046	4.241
